# Probiotic potential of *Bacillus* Isolates from Polish Bee Pollen and Bee Bread

**DOI:** 10.1007/s12602-023-10157-4

**Published:** 2023-09-19

**Authors:** Karolina Pełka, Ahmer Bin Hafeez, Randy W. Worobo, Piotr Szweda

**Affiliations:** 1 Department of Pharmaceutical Technology and Biochemistry, Faculty of Chemistry, Gdansk University of Technology, Narutowicza 11/12, 80233 Gdansk, Poland; 2 Department of Food Science, Cornell University, Ithaca, NY 14853 USA

**Keywords:** *Bacillus*, Probiotic, Bee bread, Bee pollen

## Abstract

The main goal of this study was the evaluation of the probiotic potential of 10 *Bacillus* spp. strains isolated from 5 bee bread and 3 bee pollen samples. The antagonistic interaction with *Staphylococcus aureus* and *Escherichia coli* was a primary criterion for the preliminary selection of the isolates. Three out of ten strains—PY2.3 (isolated from pollen), BP20.15 and BB10.1 (both isolated from bee bread)—were found to be possible probiotic strains. All these strains are safe for humans (exhibiting $\gamma$-hemolytic activity) and meet all essential requirements for probiotics in terms of viability in the presence of bile salts and acid conditions, hydrophobicity, auto-aggregation, and co-aggregation with the cells of important human pathogenic bacteria. They also assimilate more than 30% of cholesterol after 24 h of incubation. These three isolates are resistant to penicillin but sensitive (or exhibit moderate resistance) to the other nine antibiotics tested herein. On the basis of whole-genome sequencing, BP20.15 and BB10.1 were classified as *B. subtilis* and PY2.3 as *B. velezensis*. Moreover, genomic analyses revealed that all these isolates are potential producers of different antimicrobial compounds, including bacteriocins and secondary metabolites. The outcomes of this study have proven that some of the *Bacillus* strains isolated from bee pollen or bee bread are potential probiotics.

**Supplementary Information:**

The online version contains supplementary material available at 10.1007/s12602-023-10157-4.

## Introduction

Bee products, such as honey, propolis, or royal jelly, are known since ancient times and have been used as traditional remedies in folk medicine. Bee pollen, particularly bee bread, is still less known and less popular among consumers. However, the research conducted during the last decade indicates that both of them deserve special attention due to their wide array of health-beneficial properties such as antimicrobial, antioxidant, anti-radiation, anti-inflammatory, antitumor, hepatoprotective, and chemoprotective activity [1–5]. They are also rich sources of essential amino acids, fatty acids vitamins and microelements. Bee pollen is made up of 7–17% water, 36–37% carbohydrates (fructose and glucose), 20–23% proteins (with all necessary amino acids: methionine, lysine, threonine, histidine, leucine, isoleucine, valine, phenylalanine, and tryptophan), 5.1% fat, 2.2–3% ash content, and 1.6% phenolic compounds (flavonoids, leukotrienes, catechins, and phenolic acids) [6]. Bee workers collect pollen during plant pollination. However, it must be noted that for bees, pollen is only the raw material for preparing the final product — bee bread, which in fact is the main source of proteins for young bees and bee larvae. The pollen grains gathered from plants are mixed with a small dose of the secretion from bee workers’ salivary glands and/or nectar and placed in specific baskets (corbiculae) that are situated on the tibia of their hind legs. The bee workers transport the pollen loads to the hive, pack them in the honeycomb cells, and cover them with a thin layer of honey and a waxy lid. In these anaerobic conditions, bee pollen undergoes solid-state fermentation and biochemical changes [2, 7]. Endogenic enzymes and microflora of pollen grains, as well as enzymes and microorganisms present in bees saliva, are crucial for the biotransformation of bee pollen to bee bread [2, 3]. However, the exact mechanism of this process is not fully recognized. The most important benefits (for both bees and human consumers) of this process are (1) improvement of nutritional value of the final product (importantly higher availability of food ingredients) by partial hydrolysis of biopolymers that covers each grain of bee pollen: sporopollenin, cellulose, and pectins [8] and subsequently partial hydrolysis of proteins, lipids, and poli/oligo saccharides [8–10]; (2) stabilization of the final product against microbial spoilage that is a consequence of supplementation of bee pollen with bees glandular secretions (contains glucose oxidase and major royal jelly protein) [11], release from pollen grains polyphenols and other natural compounds that exhibit antimicrobial potential [1, 3] and first of all development of microflora producing metabolites (e.g. lactic acid and bacteriocins) that exhibit high antagonistic activity against pathogenic microorganisms [1, 2, 12–15]. Thus, the final product — bee bread is stable and safe for young bees and is a source of readily available food ingredients for bee larvae.

As mentioned above, microorganisms play a crucial role in the biotransformation of bee pollen into bee bread. Except for LAB, bacteria belonging to the genus *Bacillus* are one of the most abundant species found in bee pollen and bee bread, but also in honey [12, 16–18]. *Bacillus* spp. are widespread, spore-forming, rod-shaped bacteria that produce metabolites with biotechnological applications, including enzymes, amino acids, and antimicrobial agents [19]. In the hive, these bacteria are involved in the production of enzymes beneficial for bees’ health, the biotransformation of bee pollen into bee bread, and increasing resistance to some diseases such as chalkbrood. Sabate and colleagues (2012) reported that *B. subtilis* subsp. *subtilis* Mori2 exhibited a few benefits for bee colonies: an increase in the number of bees’ larvae, a reduction of *Varroa* and *Nosema* levels in the hive and higher honey accumulation compared to the control hives [20]. The majority of the bacterial species that belong to the genus *Bacillus* spp. are considered safe, and only a few of them, including *B. cereus* and *B. anthracis*, are human and animal pathogens [21].

The intestinal microflora plays a key role in the host body and is associated with the regulation of nutritional, immunologic, and physiological functions [22]. Disproportion of the gut microbiota can cause many gastrointestinal diseases, such as inflammatory bowel disease, obesity, or type 2 diabetes [23]. According to the WHO, probiotics are live microorganisms that, when delivered in adequate amounts, improve the host’s health. The characteristics of probiotics include the ability to tolerate gastrointestinal conditions, survivability in the presence of gastric acid and bile salts, ability to adhesion to epithelial cells, antimicrobial activity, and a lack of antibiotic-resistant genes [24]. Several strains of the genus *Bacillus* are used commercially as probiotics: *B. subtilis*, *B. polyfermenticus*, *B. clausii*, some *B. cereus*, *B. coagulans*, *B. pumilus*, and *B. licheniformis* [25]. Recent studies revealed that bee products are a good reservoir of *Bacillus* strains that were investigated in terms of probiotic potential [21, 26, 27]. Moreover, our latest study revealed that *Bacillus* strains obtained from bee pollen and bee bread demonstrated high antibacterial activity, especially against Gram-positive *staphylococci,* and produced many essential enzymes such as lipases, esterases, cellulases, and proteases [12]. In the present study, we aimed to evaluate the probiotic characteristics of selected *Bacillus* strains isolated from Polish bee pollen and bee bread.

## Materials and Methods

### Chemicals and Materials

All chemicals and reagents were purchased from commercial sources. Luria–Bertani broth (LB) was purchased from Biomaxima (Lublin, Poland); Columbia Blood Agar was bought from Graso Biotech (Starogard Gdanski, Poland); and Yeast Extract–Peptone–Dextrose (YPD) was obtained from A&A Biotechnology (Gdynia, Poland). Bile salts, phosphate-buffered saline (PBS), phenol, xylene, Mueller–Hinton Agar (MHA), DNase agar with toluidine blue, cholesterol-PEG600, o-phtalaldehyde, and Brain Heart Infusion (BHI) broth were purchased from Merck (Darmstadt, Germany). Hydrochloric acid, acetic acid, absolute ethanol, n-hexane, and sulfuric acid were bought from POCH (Gliwice, Poland), and potassium hydroxide was purchased from Chempur (Piekary Slaskie, Poland). Antibiotic disks (chloramphenicol (30 μg), azithromycin (15 μg), linezolid (30 μg), rifampin (5 μg), penicillin (10 units), trimethoprim (5 μg), clindamycin (2 μg), ciprofloxacin (5 μg), gentamycin (10 μg), and kanamycin (30 μg)) were obtained from Oxoid (Basingstoke, UK). API® CH 50 kit was bought from Biomerieux (Marcy-l'Étoile, France). Ultrapure water (18.0 Ω) was obtained with the Milli-Q Advantage A10 System (Millipore, Billerica, MA, USA).

### Bacterial and Yeasts Cultures

In this study, ten isolates from bee pollen (*n* = 5) and bee bread (*n* = 3) samples derived from Polish apiaries were isolated, cultivated, and characterized according to the protocol presented in Pełka et al. 2021 [23]. Six isolates from this study (BP20.9, BP20.15, BP15.4, BP15.1, BB10.1, and BB19.21) and four other strains isolated in our laboratory from Polish bee pollen and bee bread with interesting antimicrobial activities were selected for probiotic potential investigation. All four additional strains — namely PY2.3, PY5.3, PY6.4, and PG10.5 — effectively inhibited the growth of *S. aureus* ATCC 29213 and *E. coli* ATCC 25922 and were identified as *Bacillus* spp. based on 16S rRNA sequencing (data not published). The isolated strains were cultured on LB agar plates at 37 °C.

The frequent human pathogens, such as *Staphylococcus aureus* ATCC 29213, *S. epidermidis* ATCC 35984, *Escherichia coli* ATCC 25922, *Listeria monocytogenes* ATCC 35152, *Salmonella enterica* PCM 2266, and yeasts *Candida albicans* ATCC 10231, *C. albicans* SC 5314*, C. glabrata* DSM 6128*,* and *C. krusei* DSM 11226, were used as indicator strains for the assays aimed at determining the antimicrobial potential of the isolates. Indicator strains were cultivated on BHI agar plates (bacterial strains) and YPD agar plates (yeasts) at 37 °C.

### Acid and Bile Tolerance

To determine the survivability of potential probiotic strains in the human gastrointestinal tract (GIT), the isolates were tested in artificial gastrointestinal juices. The tolerance to low pH and the presence of bile salts were tested as described by Zulkhairi Amin et al. (2020), with slight modifications [21]. Bacterial strains were incubated in LB broth at 37 °C for 18 h at 180 rpm. The cells of the strains tested were then centrifuged (1500 s g, 10 min), washed once, and resuspended in PBS solution. Acid tolerance was tested in LB broth with pH = 2 adjusted with 0.1 M HCl, and bile salt tolerance was tested in LB broth supplemented with 0.3% bile salts. The control sample was conducted in LB broth. Into 5 ml of broth (LB, LB with pH = 2, LB with 0.3% bile salts) 50 μl of bacterial cell suspensions were added. Samples were withdrawn after time intervals (0 h and 3 h) and serially tenfold diluted using PBS. Then, the dilutions were applied to LB agar plates in triplicate. The colonies growing on the plates were counted after 24 h of incubation at 37 °C. During the 3-h interval, the samples in the particular broths were incubated at 37 °C and 100 rpm.

### Auto-Aggregation and Co-Aggregation

The adhesive properties of isolates were tested using slightly modified auto-aggregation and co-aggregation assays described by Jeon et al. (2017) [28]. Bacterial strains were incubated in LB broth at 37 °C for 18 h at 180 rpm. The isolates were then centrifuged (1500 s g, 10 min), washed once, and resuspended in PBS solution to reach an optical density of 0.1–0.2 at 600 nm (OD_600_).

To determine the auto-aggregation ability of isolates, 5 ml of bacterial suspensions were incubated at 37 °C for 4 and 24 h, and after that time, absorbance was measured at 600 nm. The percentage of auto-aggregation was calculated using the formula:

$$\left[\%\right]=(1-{A}_{t}/{A}_{0}\times 100\%$$

where A_0_ and A_t_ represented the OD_600_ at 0 h and at the indicated incubation time (4 h and 24 h, respectively).

To determine the percentage of co-aggregation, isolated strains as well as pathogen strains (*S. aureus* ATCC 29213, *E. coli* ATCC 25922, *L. monocytogenes* ATCC 35152, and *S. enterica* PCM 2266) were used. 2.5 ml of bacterial isolate suspensions were mixed with 2.5 ml of each pathogen cell suspension. The mixture was incubated at 37 °C for 4 and 24 h, and after that time, absorbance was measured at 600 nm. The percentage of co-aggregation was expressed as follows:

$$\left[\%\right]=((({A}_{P}+{A}_{B})/2)-{A}_{MIX}/(({A}_{P}+{A}_{B}/2 \times 100\%$$

where A_P_ and A_B_ represent the absorbance of the pathogen and isolated *Bacillus* strain at 0 h, respectively, and A_MIX_ represents the absorbance of the mixed culture after 4 and 24 h intervals.

### Cell Surface Hydrophobicity

To determine the potential ability of the strains to adhere to epithelial cells of the gut, the adherence to the surface of hydrocarbons (hydrophobicity assay) was measured as described by Yadav et al. (2016) [29]. Bacterial strains were incubated in LB broth at 37 °C for 18 h at 180 rpm. The cultures of the isolates were then centrifuged (1500 s g, 10 min), washed once, and resuspended in PBS solution to reach an optical density of 0.1–0.2 at 600 nm (OD_600_). 3 ml of the prepared cell suspension was mixed with 1 ml of p-xylene and incubated at 37 °C for 1 h. After aqueous and organic phase separation, 1 ml of the aqueous phase was carefully taken from the test tube, and the absorbance was measured at 600 nm. The percentage of hydrophobicity was calculated using the formula:

$$\left[\%\right]=(1- {A}_{t}/{A}_{0})\times 100\%$$

where A_0_ and A_t_ represent the absorbance of the aqueous phase after 0 and 1 h, respectively.

### Antimicrobial Activity of Isolates

Agar diffusion method was performed to investigate the antimicrobial potential of isolated *Bacillus* strains. For this assay, six bacterial and four yeast reference strains were used: *S. aureus* ATCC 29213, *S. epidermidis* ATCC 35984, *E. coli* ATCC 25922, *P. aeruginosa* ATCC 27853, *L. monocytogenes* ATCC 35152, *S. enterica* PCM 2266, *C. albicans* ATCC 10231, *C. albicans* SC 5314, *C. glabrata* DSM 6128, and *C. krusei* DSM 11226. Inoculation of agar media LB (for bacteria) and YPD (for yeasts) was performed by streaking with a sterile cotton swab soaked in a suspension of each tested indicatory strain (final optical density of each suspension OD_600_ = 0.1). Subsequently, the colonies of isolates from the collection were transferred with sterile pipette tips in the form of dots onto LB agar plates (for bacteria) and YPD agar plates (for yeasts). Afterward, plates were incubated at 37 °C for 24 h. After incubation, growth inhibition zones of indicatory strains around colonies (dots) of isolates were observed and measured.

### Antibiotic Disk Susceptibility Test

According to the Performance Standards for Antibiotic Disk Susceptibility Tests (CLSI)**,** the antibiotic susceptibility assay was performed on MHA plates using the antibiotic disk diffusion method. The different groups of antibiotics were selected according to their different modes of action: protein synthesis inhibition (chloramphenicol, azithromycin, linezolid, clindamycin, gentamicin, kanamycin), cell wall synthesis inhibition (penicillin), and DNA or RNA synthesis inhibition (rifampin, ciprofloxacin, trimethoprim). The following antibiotic disks were used: chloramphenicol 30 μg, azithromycin 15 μg, linezolid 30 μg, rifampin 5 μg, penicillin 10 units, trimethoprim 5 μg, clindamycin 2 μg, ciprofloxacin 5 μg, gentamicin 10 μg, and kanamycin 30 μg. Bacterial cell suspensions (0.5 McFarland) were prepared, spread on MHA plates, and allowed to dry. Then, the antibiotic disks were placed on plates and incubated at 37 °C for 18 h. After incubation, the diameters of the inhibition zones were measured. The results were compared with interpretative zone diameters described by the Performance Standards for Antibiotic Disk Susceptibility Tests (CLSI). The criteria used for the interpretation of the results were adapted from the M100 Performance Standards determined for *Staphylococcus* spp. [30].

### Hemolytic Activity

To examine the safety of isolates, the hemolytic activity test was performed using Columbia Agar plates with 5% sheep blood. Overnight-grown isolate cultures were streaked onto blood agar plates and incubated at 37 °C for 24 h. Thereafter, the zones of hemolysis around the grown colonies were observed.

### DNase Activity

For examination of DNase production by isolates, DNase medium with toluidine blue was used. Overnight-grown isolate cultures were streaked onto DNase agar plates and incubated at 37 °C for 24 h. After incubation, the change in color of the DNase medium around the grown colonies was observed.

### In vitro Cholesterol Assimilation

Cholesterol assimilation analyses were performed according to the protocol described by Tomaro-Duchesneau and colleagues (2014) [31], with some modifications. Filter-sterilized water-soluble cholesterol solution was added to LB broth with 0.2% ox gall bile at a final concentration of 100 μg/ml and inoculated with 1% (*v/*v) bacterial overnight culture. Afterward, the suspension was incubated at 37 °C for 24 h. After incubation, the suspension was centrifuged (5500 rpm, 7 min, 4 °C), and the supernatant was collected. 0.5 ml of supernatant was mixed with 1 ml of absolute ethanol and 0.5 ml of 33% (w/v) KOH. The mixture was vortexed and incubated at 37 °C for 15 min. After cooling the samples to room temperature, 1 ml of deionized water and 1.5 ml of n-hexane was added to the solutions and vortexed for 1 min. Afterward, the mixture was left at ambient temperature for phase separation. Subsequently, 0.5 ml of the upper n-hexane phase was removed and transferred into a new test tube, and then evaporated at 45 °C using a rotary concentrator (Eppendorf Concentrator Plus, Hamburg, Germany). Subsequently, 1 ml of o-phtalaldehyde reagent (50 mg/dl in acetic acid) was added into tubes and mixed. Then, 250 μl of concentrated sulfuric acid was added, vortexed for 1 min and allowed to stand for 20 min at room temperature. The absorbance of the final mixture was measured using TECAN Multiplate Reader (Spark 10M Grödig, Austria) at 570 nm. For calculations, a standard curve of cholesterol was prepared for cholesterol concentrations from 0 to 500 μg/ml in LB broth. Cholesterol reduction was calculated as follows:

$$\mathrm{assimilated cholesterol} \left[\%\right]=(({CH}_{NC}- {CH}_{s})/{CH}_{NC})\times 100$$

where CH_NC_ represents the amount of cholesterol present in the negative control (non-inoculated broth) and CH_S_ represents the amount of cholesterol present in samples.

### Carbohydrates Metabolism by Isolated Strains

For determining the metabolism of carbohydrates, API® ZYM and API® 50 CH kits were used, respectively. Performance of the assays and analysis of the results were conducted according to the protocols described by the manufacturer.

### DNA Extraction and Whole-Genome Sequencing Analysis

DNA Sequencing and Oligonucleotide Synthesis Laboratory, Institute of Biochemistry and Biophysics, Polish Academy of Science (Warsaw, Poland), performed the genomic DNA extraction and sequencing. The CTAB/lysozyme method was used, cell pellets from the overnight LB culture were treated, and the quality and quantity of template DNA were checked on the agarose gel. Following the manufacturer’s instructions, KAPA Library Preparation Kit (KAPA/Roche, Basel, Switzerland), the genomic DNA was sheared to an appropriate size for Paired-End TruSeq-like library construction. The bacterial genomes were sequenced in paired-end mode (V3,600 cycle chemistry kit) using MiSeq (Illumina, San Diego, CA, USA).

FASTQC was used to assess the quality of raw reads. The reads were trimmed and paired using Trimmomatic (version 0.38.0) [32] with the following parameters: LEADING: 3 TRAILING: 3 SLIDINGWINDOW: 4:20 MINLEN: 28. To ensure normal results for “per base sequence quality,” “per base N content,” “sequence duplication levels,” and “adapter content,” a secondary read quality check was performed. De novo assembly was performed with SPAdes (3.15.5) with the parameters: -k 33, 55, 77, 99, 127 -careful. Scaffolds less than 500 bp were removed, and assembly statistics [e.g., number of contigs, N50 (widely used to assess the contiguity of an assembly), G + C content] were assessed using QUAST (Version 5.0.2) [33]. The final assemblies were BLASTed in the National Center for Biotechnology Information (NCBI) database, and the genome assemblies of closely related group-type strains were downloaded from the assembly database. The orthoANI method using OAT (version 0.93.1) and BLAST + (version 2.13.0) was used to calculate the average nucleotide identity (ANI) of the isolates [34]. The genomes of strains BB10.1, BP20.15, and PY2.3 were compared to the respective type strains with Mauve (v20150226) using progressive alignment and seed-families options [35]. Rapid genome annotation was performed using Prokka (version 1.14.6) [36]. Additional genome annotation was performed with the NCBI Prokaryotic Genome Annotation Pipeline (PGAP) (https://github.com/ncbi/pgap) on the local machine [37].

Assembled genomes of the strains BB10.1, BP20.15, and PY2.3 were submitted to the Sequence Read Archive (SRA) and GenBank under the BioProject IDs PRJNA949953, PRJNA949979, and PRJNA949984, respectively. SRA accession numbers for 10.1, 20.15, and PY2.3 are SRR24003043, SRR24003725, and SRR24005238, respectively.

Moreover, the bacteriocin-encoding gene clusters were identified with the BAGEL4 server.

### Phylogenomics Analysis

The whole-genome-based taxonomic analysis was performed by the Type (strain) Genome Server (TYGS) (https://tygs.dsmz.De) [38]. The draft genomes of isolates BB10.1, BP20.15 and PY2.3 sequenced in this study and 22 other closely related genomes of the *B. velezensis* and *B. subtilis* groups, as well as the complete genome of *B. cereus* ATCC 14579 strain as an outgroup, were extracted from NCBI and submitted to the TYGS server, settings: restricted genome mode. A phylogenomic tree was constructed with FastME (based on balanced minimum evolution and renders distance algorithms to infer phylogenies) [39] using the genome blast distance phylogeny (GBDP) method and annotated using Interactive Tree of Life (iTOL) v5, an online tool for phylogenetic tree display and annotation [40]. All pair-wise genome comparisons were carried out with GBDP and inter-genomic distances inferred under the algorithm “trimming” and distance formula d5 [38]. The tree was rooted at the midpoint [38]. Branch supports were inferred from 100 pseudo-bootstrap replicates.

### Statistical Analysis

All experiments were performed in triplicate. The results are expressed as means ± standard deviation (SD) of the mean and checked for normality using D’Agostino-Pearson normality test. Linear regression was carried out for standard curve formation. The data were analyzed using GraphPad Prism (ver. 9.4.1). A statistical comparison was conducted using a two-way ANOVA followed by Tukey’s multiple comparison tests. *p* < 0.05 was considered significant.

## Results

### Strains Survivability in the Presence of Acid and Bile Salts

The ability to survive in simulated gastrointestinal conditions is presented in Table 1. After exposure of bacterial cells to acidic conditions and to a 0.3% bile salt solution, the survivability of isolates was in the range of 35.50–68.23% and 84.63–110.15%, respectively. BB19.21 and BP20.9 had the highest survival rates in the harsh conditions of GIT among all strains tested, whereas strains BP5.3 and PG10.5 demonstrated the weakest survivability in the tested conditions.

**Table 1:** Comparison of percentage viability of strains in LB broth (growth control), and LB broth with 0.3% bile salts and acid (pH = 2) after the indicated time of incubation. The assay was performed in triplicate and the results are presented as mean values ± SD. ^a – d^ – different superscript letters represent statistical differences between strains at the level of *p* < 0.05 measured by Tukey’s test. The superscript letter (a) describes the highest significance, (d) – the lowest significance, and (e) – no significant difference between samples

	**Growth control (3h) [%]**	**Bile salts tolerance (3h) [%]**	**Acid tolerance (3h) [%]**
PY2.3	122.33 ± 4.46^c,e^	87.10 ± 2.17^a,d,e^	68.23 ± 0.68^a,d,e^
PY5.3	108.31 ± 0.79^b,d,e^	84.63 ± 0.44^a,c,d,e^	50.14 ± 3.06^a,b,c,e^
PY6.4	116.42 ± 0.86^e^	90.51 ± 0.80^a,b,e^	56.97 ± 2.08^a,d,e^
PG10.5	115.77 ± 1.83^e^	99.28 ± 1.30^a,c,d^	35.50 ± 4.24^a,c,d^
BP20.9	116.01 ± 1.43^e^	108.87 ± 0.95^a,c,d,e^	64.02 ± 2.75^a,b,c,e^
BP20.15	119.88 ± 0.06^d,e^	95.63 ± 1.22^c,d,e^	47.89 ± 4.94^a,b,d,e^
BP15.4	119.96 ± 1.90^d,e^	97.25 ± 0.67^c,d,e^	58.04 ± 2.28^a,e^
BP15.1	116.79 ± 5.42^e^	95.53 ± 0.28^c,d,e^	65.89 ± 1.72^a,b^
BB10.1	120.73 ± 3.86^d,e^	107.81 ± 0.31^a,b,d,e^	57.53 ± 1.58^a,e^
BB19.21	124.27 ± 5.42^b,e^	110.15 ± 1.39^a,c,d,e^	65.88 ± 3.07^a,b,e^

### Surface Properties of Isolated Strains

The possible adhesive properties of isolates are presented in Table 2. To determine the hydrophobicity of isolates, the adhesion of bacterial cells to xylene was examined. Hydrophobicity varied from 5.69 to 61.08%, while the maximum affinity toward xylene was exhibited by strain PG10.5 and the minimum by BB19.21.

**Table 2:** Adhesive properties of isolated strains from BP and BB. The assay was conducted in triplicate. The percentages of hydrophobicity and auto-aggregation are expressed as mean values ± SD. ^a – d^ – different superscript letters represent statistical differences between strains at the level of *p* < 0.05 measured by Tukey’s test. The superscript letter (a) describes the highest significance, (d) – the lowest significance, and (e) – no significant difference between samples

	**Hydrophobicity [%]**	**Auto-aggregation**	
		**After 4 h [%]**	**After 24 h [%]**
**PY2.3**	22.72 ± 1.37^a,b,c,d,e^	38.62 ± 2.14^a,b,e^	87.30 ± 3.37^a,e^
**PY5.3**	22.66 ± 1.09^a,b,c,d,e^	38.66 ± 2.77^a,b,e^	87.42 ± 2.89^a,e^
**PY6.4**	8.36 ± 2.11^a,b,e^	21.55 ± 0.04^a,c,e^	61.70 ± 3.66^a,b,e^
**PG10.5**	61.08 ± 2.19^a^	27.34 ± 2.00^a,b,c,e^	50.06 ± 2.92^a,b,c,e^
**BP20.9**	10.71 ± 1.62^a,c,e^	25.02 ± 1.86^a,b,e^	47.08 ± 1.32^a,b,e^
**BP20.15**	33.18 ± 1.65^a,d,e^	46.75 ± 0.28^a,e^	88.52 ± 0.95^a,e^
**BP15.4**	17.40 ± 1.38^a,c,e^	25.13 ± 0.89^a,b,c,e^	65.12 ± 2.16^a,b,c,e^
**BP15.1**	10.63 ± 2.59^a,c,e^	31.62 ± 3.16^a,c,d,e^	68.74 ± 3.09^a,c,d,e^
**BB10.1**	26.19 ± 0.87^a,e^	38.83 ± 1.05^a,b,c,e^	86.30 ± 1.30^a,b,c,e^
**BB19.21**	5.69 ± 0.81^a,c,e^	22.82 ± 1.16^a,c,d,e^	48.55 ± 0.84^a,c,d,e^

**Fig. 1:**
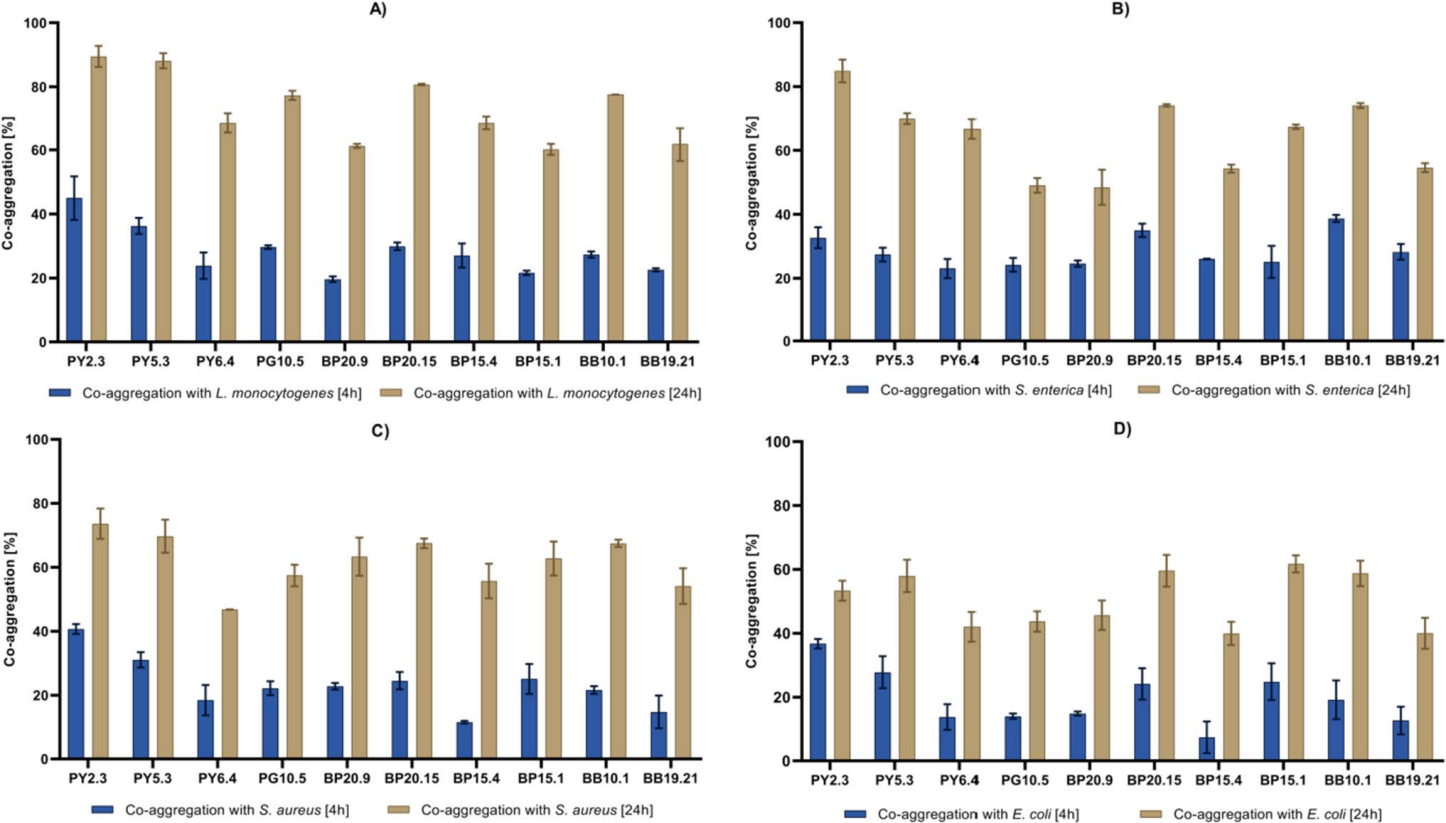
Percentage of co-aggregation of *Bacillus* isolates with **A** *L. monocytogenes* ATCC 35152, **B** *S. enterica* PCM 2266, **C** *S. aureus* ATCC 29231, and **D** *E. coli* ATCC 25922 after 4 and 24 h of incubation. The results are presented as means ± SD (*n* = 3). Data without error bars indicates that SD is too small to be observed on the graph

Auto-aggregation of isolates was in the range of 21.55–46.75% after 4 h of incubation and 47.08–88.52% after 24 h of incubation. Strain BP20.15 showed the highest ability to aggregate, followed by BB10.1. The lowest percentage of auto-aggregation was exhibited by strains BP6.4 and BB19.21. A co-aggregation (Fig. 1.) assay was performed using four pathogenic bacterial strains: *S. aureus* ATCC 29231, *E. coli* ATCC 25922, *L. monocytogenes* ATCC 35152, and *S. enterica* PCM 2266. All strains tested exhibited the best co-aggregation ability with *L. monocytogenes*; it ranged from 60.20% (BP15.1) to 89.54% (PY2.3) after 24 h of incubation. Slightly worse co-aggregation was observed for *S. enterica* and *S. aureus*. The percentage of co-aggregation of *Bacillus* isolates with these strains was from 48.47% (BP20.9) to 84.91 (PY2.3) and from 46.76% (PY6.4) to 73.63 (PY2.3), respectively. Tested isolates demonstrated the weakest co-aggregation ability with *E. coli*, from 39.94% (BP15.1) to 59.59% (BP20.15).

### Antimicrobial Activity of Bacillus Isolates

Isolated strains were tested for antimicrobial activity against bacterial and yeast pathogens, results presented in Table 3. The antimicrobial activity against pathogens cultivated on agar plates was determined by measuring the diameter of inhibition zones around the growing colonies of isolates. The most sensitive bacterial strains were Gram-positive *staphylococci*. Nine and ten out of ten tested isolated strains inhibited the growth of *S. aureus* and *S. epidermidis*, respectively. On the other hand, the most resistant Gram-positive bacteria tested was *L. monocytogenes*. Among Gram-negative bacterial pathogens, *P. aeruginosa* was the most sensitive strain; eight out of 10 isolates inhibited the growth of this strain. Moderate sensitivity was exhibited by *E. coli* and *S. enterica*. Five and six isolates, respectively, inhibited the growth of these pathogens on agar plates. Furthermore, isolated *Bacillus* strains showed reasonable activity against two *C. albicans* strains. Growth of *C. albicans* SC 5314 was inhibited by seven out of ten tested strains, and growth of *C. albicans* ATCC 10231 was inhibited by five strains. Only one strain, BP20.9, was able to slightly inhibit *C. krusei* growth.. None of the tested isolates demonstrated antifungal activity against *C. glabrata*.

**Table 3:** Antagonistic interactions of isolates with pathogenic bacteria and yeasts. ( +) – inhibition zone with diameter 1–2 mm, (+ +) – 3–4 mm, (+ + +)—≥ 5 mm, (-) – no inhibition zones around growing colonies

	***S. aureus* ATCC 29213**	***S. epidermidis* ATCC 35984**	***E. coli* ATCC 25922**	***P. aeruginosa* ATCC 27853**	***S. enterica* PCM 2266**	***L. monocytogenes A*TCC 35152**	***C. albicans* SC 5314**	***C. albicans* ATCC 10231**	***C. krusei* DSM 11226**	***C. glabrata* DSM 6128**
PY2.3	+ +	+ +	+	+ + +	+	-	-	-	-	-
PY5.3	+ +	+ +	+	+ + +	+	-	+ +	+	-	-
PY6.4	+	+	-	+ +	+	-	-	-	-	-
PG10.5	+	+	-	-	+	+ +	-	-	-	-
BP20.9	+ + *	+ +	+ + *	+ + *	-	-	+	+	+	-
BP20.15	+ + *	+	-*	+ + + *	-	-	+	-	-	-
BP15.4	+ + + *	+ + +	+ + + *	+ + *	+	+	+	+	-	-
BP15.1	-*	+	-*	+ *	+	-	+	+	-	-
BB10.1	+ + + *	+ +	-*	+ + + *	-	-	+	-	-	-
BB19.21	+ + + *	+ +	+ + + *	+ + + *	-	-	+	+	-	-

*—results presented in our previous paper (Pełka et al., 2021b)

### Antibiogram of Isolated Strains from BP and BB

The sensitivity of isolates to particular antibiotics is presented in Table 4. The high susceptibility of all isolated strains was observed against chloramphenicol, azithromycin, linezolid, trimethoprim, ciprofloxacin, gentamicin, and kanamycin. However, six strains were resistant to penicillin and two to clindamycin. The rest of the tested antibiotics presented moderate activity against isolates.

**Table 4:** Results of Antibiotic Disk Susceptibility Test. In the table below, the diameter of inhibition zones around disks are presented (in mm). The superscript letter (S) represents high sensitivity to antibiotics, (M) – moderate susceptibility, and (R) – resistance to tested antibiotics (according to the CLSI Standard). C30—chloramphenicol (30 μg), AZM15—azithromycin (15 μg), LZD30—linezolid (30 μg), RD5—rifampin (5 μg), P10—penicillin (10 units), W5—trimethoprim (5 μg), DA2—clindamycin (2 μg), CIP5—ciprofloxacin (5 μg), CN10—gentamicin (10 μg), and K30—kanamycin (30 μg)

	**C30**	**AZM15**	**LZD30**	**RD5**	**P10**	**W5**	**DA2**	**CIP5**	**CN10**	**K30**
PY2.3	24^S^	17^M^	28^S^	18^M^	21^R^	23^S^	20^M^	27^S^	18^S^	21^S^
PY5.3	25^S^	19^S^	26^S^	18^M^	22^R^	24^S^	21^S^	30^S^	20^S^	26^S^
PY6.4	14^M^	19^S^	30^S^	30^S^	22^R^	29^S^	0^R^	35^S^	19^S^	23^S^
PG10.5	22^S^	19^S^	29^S^	22^S^	34^S^	36^S^	19^M^	33^S^	18^S^	21^S^
BP20.9	22^S^	20^S^	31^S^	23^S^	35^S^	35^S^	17^M^	35^S^	19^S^	21^S^
BP20.15	29^S^	23^S^	29^S^	17^M^	23^R^	26^S^	21^S^	33^S^	19^S^	23^S^
BP15.4	20^S^	19^S^	30^S^	19^M^	33^S^	33^S^	17^M^	30^S^	19^S^	22^S^
BP15.1	24^S^	27^S^	33^S^	22^S^	22^R^	27^S^	0^R^	35^S^	19^S^	22^S^
BB10.1	27^S^	18^S^	30^S^	17^M^	24^R^	30^S^	18^M^	29^S^	28^S^	20^S^
BB19.21	23^S^	21^S^	28^S^	21^S^	37^S^	33^S^	18^M^	32^S^	17^S^	20^S^

### Hemolytic and DNase Activity of Isolates

Hemolytic and DNase activities of the tested strains were examined. There were no pinkish zones around growing colonies. Thus, none of them demonstrated DNase activity. In terms of ability to hemolyze, two strains (BP20.9 and BP15.4) presented β-hemolytic activity, three showed α-hemolytic activity (PY5.3, BB19.21, and PG10.5), and the rest of the tested isolates exhibited γ-hemolytic activity, which is considered safe for humans.

### Cholesterol Assimilation of Isolates

**Fig. 2:**
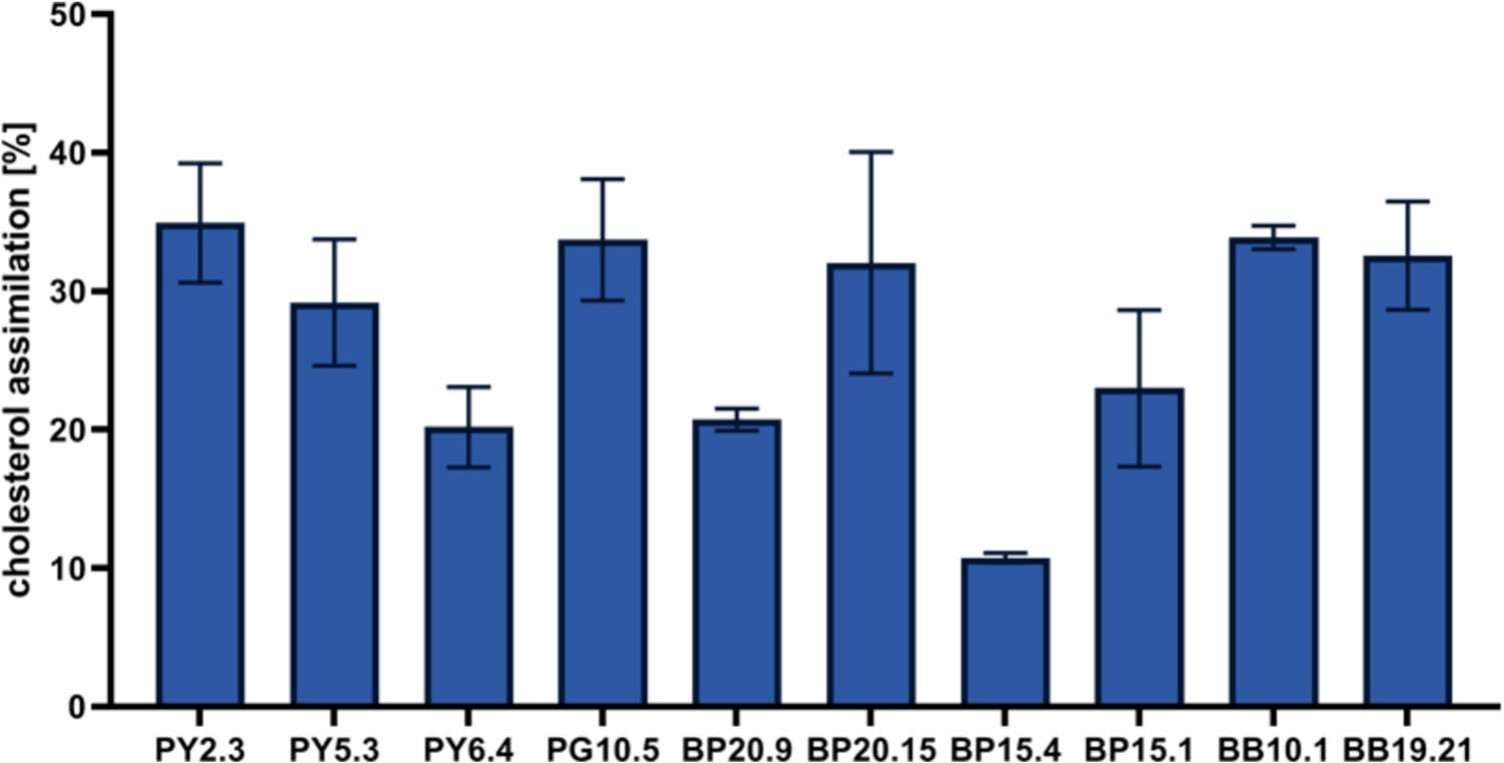
The amount of cholesterol assimilated by isolates [%] after 24 h of incubation. The results are presented as means ± SD (*n* = 3)

The ability to assimilate cholesterol in LB broth supplemented with 0.2% of bile salts was determined. The mean value of cholesterol concentration assimilated by strains after 24 h was 27.99 ± 3.80%. The highest cholesterol absorption was exhibited by strain PG10.5 (36.45 ± 4.65%) and the lowest by strain BP15.4 (10.74 ± 0.240%) Fig. 2.

### Carbohydrates Metabolism and Profile of Enzymes Produced by Strains

Isolates were also investigated according to their ability to metabolize carbohydrates using the API CH50 kit.

Tested strains were able to metabolize 30 out of 49 carbohydrates (Supplementary information, Table 1). The most abundant carbohydrates processed by tested strains were glycerol, L-arabinose, D-ribose, D-xylose, D-glucose, D-fructose, D-mannose, D-mannitol, methyl-α-D-glucopyranoside, amygdalin, arbutin, esculin, salicin, D-cellobiose, D-maltose, D-saccharose, D-trehalose, amidon, glycogen, and D-tagatose. On the other hand, none of the investigated strains metabolized erythritol, D-arabinose, L-xylose, D-adonitol, methyl-β-D-xylopyranoside, L-sorbose, L-rhamnose, dulcitol, methyl-α-D-mannopyranoside, D-melezitose, xylitol, D-lyxose, D-fucose, D-arabitol, L-arabitol, potassium gluconate, potassium 2-ketogluconate, and potassium 5-ketogluconate.

### Whole-genome Sequencing Analysis

The 4,051,332 bp genome of isolate BB10.1 was assembled into 24 contigs with a GC content of 43.67% and an N50 of 1,041,549 bp (Supplementary Table 2, Quast report). The 4,022,011 bp genome of isolate BP20.15 was assembled into 53 contigs with a GC content of 43.71% and an N50 of 185,768 bp (Supplementary Table 2, Quast report). The genome of isolate PY2.3 (3,913,508 bp) was assembled into 35 contigs with a GC content of 46.52% and an N50 of 353,859 bp (Supplementary Table 2, Quast report). Isolate BB10.1 contained an estimated 4241 genes and 4138 coding sequences (CDSs), 103 RNAs, 14 rRNAs (12; 5S, 1; 16S, and 1; 23S) and 84 tRNAs. Isolate BP20.15 consisted of an estimated 4207 genes, 4122 CDSs, 85 RNAs, one rRNA (5S) and 79 tRNAs. On the other hand, isolate PY2.3 had 3891 genes, 3807 CDSs, 84 RNAs, three rRNAs (5S, 16S and 23S) and 76 tRNA.

Average nucleotide identity (ANI) classified the isolates at the species level. Isolates BB10.1 and BP20.15 were most closely related to type strains *B. subtilis* Strain168 *and B. subtilis* 75, with orthoANI values of 99.97% and 99.99%, respectively. Isolate PY2.3 was closely related to *Bacillus velezensis* S4 with an orthoANI value of 98.69% (Table 5). The ANI between isolates 10.1 and 20.15 was 98.77%. Based on the proposed species boundary of 95–96% orthoANI value, 10.1 and 20.15 were classified as *B. subtilis* and PY2.3 as *B. velezensis* [41, 42].

**Table 5:** Average nucleotide identity (ANI) calculated by OAT with BLAST +

Query genome	Reference genome	OrthoANI value (%)
Isolate 10.1	*Bacillus subtilis* Strain168	99.97
Isolate 20.15	*Bacillus subtilis* 75	99.99
Isolate 10.1	Isolate 20.15	98.77
Isolate PY2.3	*Bacillus velezensis* S4	98.69
Isolate PY2.3	*Bacillus velezensis* NZ4	98.23

### Bacteriocin-encoding Genes

**Fig. 3:**
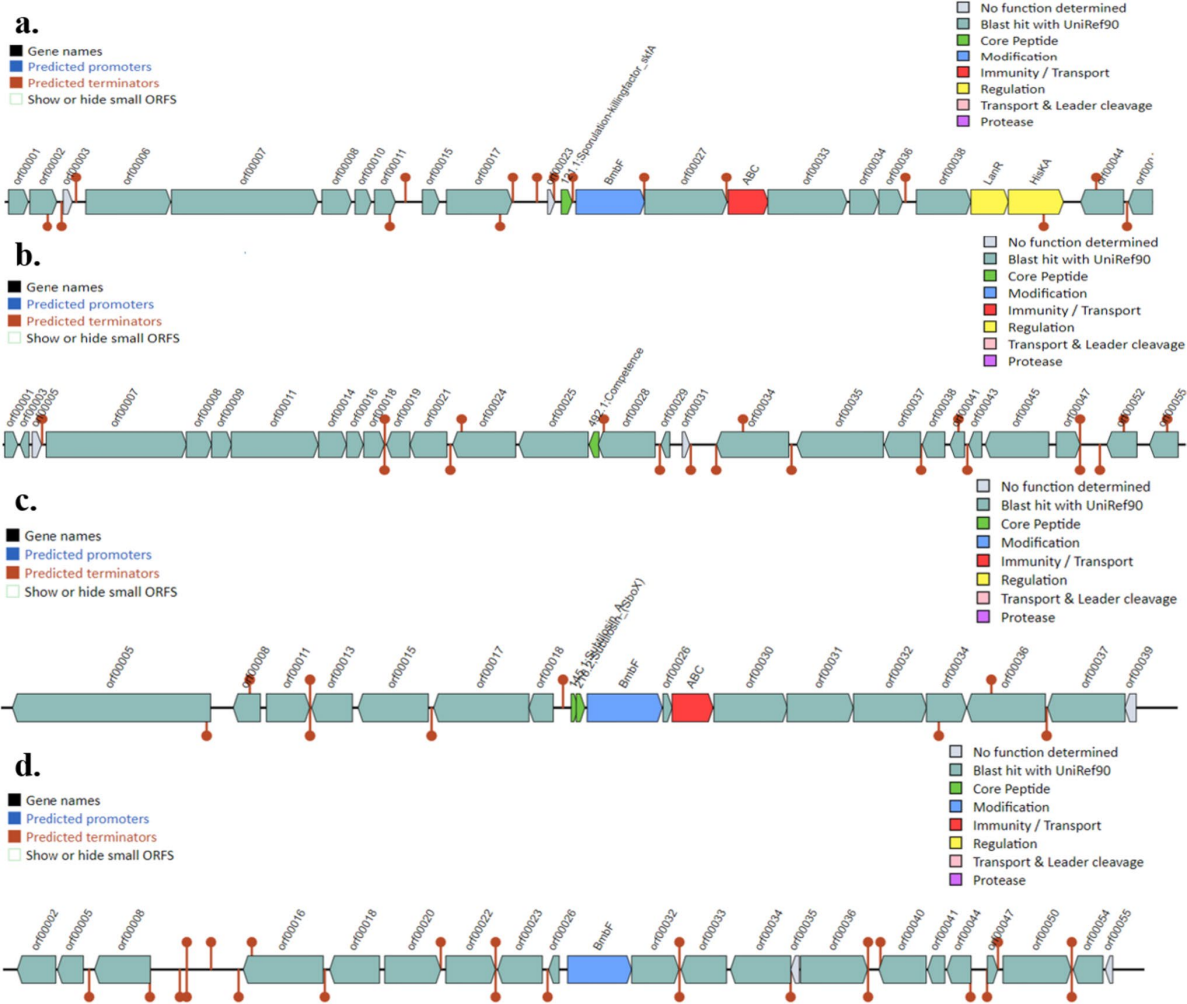
The organization of bacteriocin gene clusters in the isolate *B. subtilis* 10.1 genome predicted through the BAGEL4 webserver. The area of interests represents (a) sporulation killing factor (b) competence pheromone (c) subtilosin (d) sactipeptide classes. The color schemes represent the specific gene clusters identified in BB10.1 genome

The blast results of the BAGEL4 webserver for isolate BB10.1 genome predicted four bacteriocin clusters as areas of interest (AOIs) at (i) AOI 7.0 (start at 27,755 and end at 48,325), (ii) AOI 3.5 (start at 67,414 and end at 87,576), (iii) AOI 3.5 (start at 647,654, end at 668,287), and (iv) Node 4.7 (start at 67,715 and end at 87,715). The AOI 7.0 encodes the sporulation-killing factor *skfA*, which resides beside the *bmbF* gene, ABC transporter, ATP-binding proteins, and several ORFs (Open Reading Frames) (Fig. 3). The AOI 3.5, encodes a competence peptide that is found in several ORFs. Another AOI 3.5 was found to code for subtilosin A and subtilosin (*sboX*), which reside near the *bmbF* gene, ABC genes, and several ORFs. The AOI 4.7 encodes a bacteriocin belonging to the sactipeptide class (ribosomally synthesized and post-translationally modified peptides), which consists of a *bmbF* gene and several ORFs. Similarly, in the isolate BP20.15 genome, the same four clusters as in isolate 10.1 were observed: (i) AOI 7.1 Sporulation-Killing factor *skfA* (start: 163,457, end: 184,029); (ii) AOI 3.25 sactipeptides (start: 13,277, end: 33,277); (iii) AOI 8.37 competence (start: 123,287, end: 143,407); (iv) AOI Subtilosin (*sboX*) (start: 196,376, end: 217,009) (supplementary figure S1). In the isolate PY2.3 genome, four bacteriocin clusters were predicted as areas of interest (AOIs) at (i) AOI 5.11 (start: 122,123, end: 142,258), (ii) AOI 7.12 (start: 109,859, end: 130,189), (iii) AOI 7.12 (start: 59,723, end: 79,891), and (iv) AOI 6.5 (start: 180,338, end: 200,338). The AOI 5.11 encodes the antimicrobial peptide LCI, which resides alongside several ORFs. The AOI 7.12 encodes for amylocyclicin, which is located near a lantibiotic ABC transporter ATP-binding protein with several ORFs. Another AOI, 7.12, coded for a competence pheromone and is located between several ORFs. AOI 6.5 encodes a bacteriocin belonging to the sactipeptide class (ribosomally synthesized and post-translationally modified peptides), which consists of the *bmbF* gene and several ORFs (supplementary figure S2).

### Phylogenomics Analysis

**Fig. 4:**
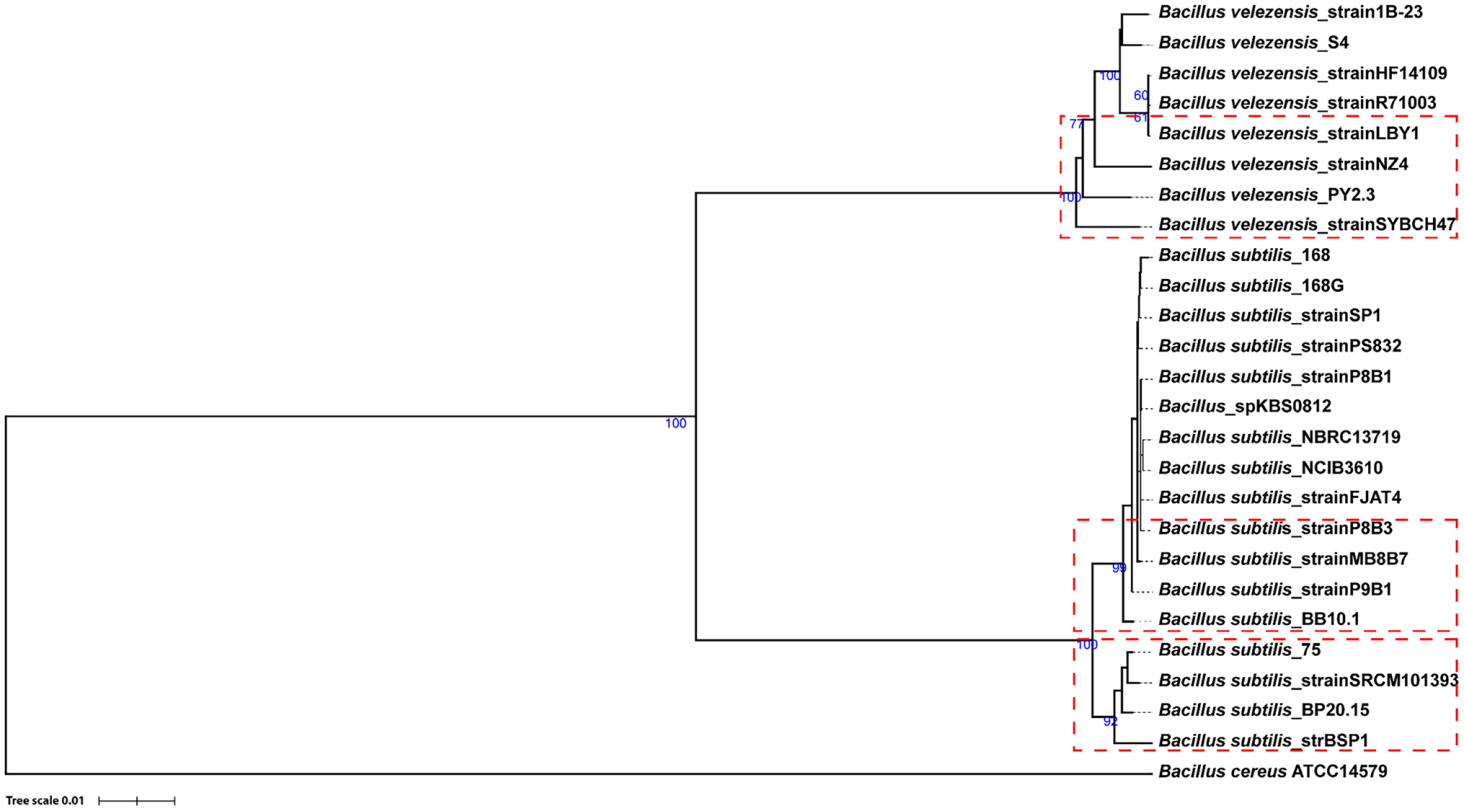
(A). TYGS Genome tree inferred with FastME 2.1.6.1 [39] from GBDP distances calculated from genome sequences. The branch lengths are scaled in terms of the GBDP distance formula d5. The numbers above branches are GBDP pseudo-bootstrap support values > 60% from 100 replications, with an average branch support of 21.2%. The tree was rooted at the midpoint [43]

To elucidate the phylogenetic relationships between our isolates and the closely related *Bacillus* species, a total of 23 genomes were downloaded from the NCBI database. Seven *B. velezensis* group isolates, 15 *B. subtilis* isolates, and one *B. cereus* ATCC 14579 as an outgroup were included in the phylogenetic analysis. The phylogenetic tree based on the entire genome revealed that the isolates from this study have relatedness to the type strains *B. subtilis* (isolates BB10.1 and BP20.15) and *B. velezensis* (isolate PY2.3) (Fig. 4).

## Discussion

Diet plays a key role in our physical and mental health. It is not only a source of essential chemical ingredients such as proteins, carbohydrates, fatty acids, vitamins, and many other micro- and macroelements but also of different strains of microorganisms. Some of the food-associated bacteria (e.g., *Salmonella, Listeria,* and *Staphylococci*) and fungi (e.g., molds of the genus *Aspergillus* and Fusarium) are dangerous pathogens. Different technological approaches are available and proposed for growth inhibition (e.g., cooling and freezing) and elimination (sterilization, high pressure, smoking, and acidification) of pathogenic microorganisms from food products. Other food-associated microorganisms called probiotics are beneficial for different aspects of our health. The best-known group of probiotics are lactic acid bacteria (LAB) that are provided to our body mostly with fermented milk products (yogurt and kefir) and fermented vegetables (e.g., cabbage and cucumbers). This study aimed to investigate the probiotic potential of bacterial strains isolated from bee pollen and bee bread. Bee bread is also an example of a fermented food product. It is generated in the wells of honeycombs during the fermentation process of bee pollen. Bacteria sourced from raw materials (pollen grains) and bee saliva play a crucial role in the process of biotransformation (fermentation) of bee pollen into bee bread. Still, very little is known about the microbiota of bee bread and pollen grains and their potential influence on the health of consumers. However, several authors reported that the microbial composition of maturing bee bread is dynamic and is changing over time. Vasquez and Olofsson (2015) observed the intense growth of LAB within maturing BB for about two weeks—the first step of BP biotransformation [13]. Disayathanoowat and coworkers (2020) found a significant decrease in the number of pathogenic Enterobacteriaceae (*Escherichia, Shigella, Panteoa,* and *Pseudomonas*) in maturing bee bread in less than 72 h from the time of pollen grain collection in honeycomb wells [44]. This observation is important not only from the point of view of the health of the bee colony but also from the perspective of the possibility of using bee pollen and/or bee bread as a food product for humans. Our previous report revealed that most of the bacteria isolated from bee pollen (partially dried) and mature bee bread belonged to the genus *Bacillu*s spp., and many of them exhibit promising antimicrobial potential [12]. Herein, we investigated the probiotic potential of selected *Bacillus* spp. strains derived from bee pollen and bee bread produced in Polish apiaries.

The probiotic bacteria are expected to improve the health of consumers mostly by regulating the microflora of the gastrointestinal tract and eliminating pathogenic bacteria. The gastrointestinal tract can be a hostile environment for microorganisms. Thus, it was important to examine the resistance of isolated strains (potential probiotics) to the bile salts and low pH. All tested isolates exhibited significant tolerance to 0.3% of bile salt with a viability level of about 80%, a concentration that is considered similar to human bile juice [45]. A slightly higher inhibitory effect was observed in acidic conditions. About 65% of the cells of the most sensitive PG10.5, were eliminated from the suspension within 3 h of incubation. However, for 8 out of 10 strains tested, the survival rate was higher than 50%, with the highest value of 68.23% for PY2.3. The properties of these strains are similar to those of bee products-derived bacteria investigated by other authors. For instance, a low influence of bile salts (at a concentration of 0.3%) on *Bacillus* spp. strains isolated from honey was observed by Toutiaee et al. (2022) [26] and Zulkhairi Amin et al. (2019) [21]. However, both strains investigated by Zulkhairi Amin et al., (2019) and three out of five strains investigated by Toutiaee et al. (2022) exhibited a bit better tolerance to acidic conditions (pH 2.0 or 3.0, respectively), with a survival rate of about 90%. Other essential abilities for potential probiotics are hydrophobicity and auto-aggregation, which reflect cell adhesion to intestinal epithelial cells [46]. The auto-aggregation ability of the strains tested herein was similar to that observed by Toutiaee et al. (2022) [26] and Zulkhairi Amin et al., (2019) [21], where the values of this parameter were in the range of 42 to 84%. Interestingly, the hydrophobicity of our nine isolates was in the range of 5.65 to 33.18%, and only one strain was above 60%, whereas the hydrophobicity of strains tested in the above-mentioned studies was in the range of 48 to 68%, thus being significantly higher. However, a similar level of hydrophobicity was observed for *B. subtilis* Bn1 by Nithya and colleagues (2013) [47]. Considering these “parameters,” we can conclude that the *Bacillus* spp. strains investigated in this study meet the basic criteria of probiotics. Moreover, interesting and beneficial results were observed for co-aggregation ability with selected pathogenic bacteria. All tested strains exhibited the highest level of co-aggregation with *L. monocytogenes,* which can cause listeriosis [48]. The values of this parameter, when tested against other pathogenic strains, were also at a satisfactory level.

One of the most important requirements for bacteria to be considered probiotics is that they are safe for the human body. Antibiotic susceptibility tests showed that all strains tested were sensitive to five out of ten antibiotics with different mechanisms of action. Chloramphenicol and azithromycin also exhibited good activity; in both cases, only one isolate with moderate susceptibility was identified (nine were sensitive). Five strains exhibited moderate resistance to rifampicin; six, namely, PY2.3, PY5.3, PY6.4, BP20.15, BP15.1, and BB10.1, were resistant to penicillin (10 u), and two isolates (PY6.4 and BP15.1) were resistant to clindamycin (2 μg). Six strains exhibited moderate susceptibility to this antibiotic. In the case of probiotics, resistance to antibiotics should be rather considered an advantage; e.g., some commercially available probiotic strains, e.g., *Lactobacillus rhamnosus* GG, also exhibit resistance to some antibiotics (teicoplanin and vancomycin) [21]. It enables the use of these bacterial strains or even bee pollen/bee bread (carriers of probiotic strains) for the regulation of the microbiota of our gastrointestinal tract during therapies conducted with these antibiotics. Another important aspect investigated was hemolytic and DNase activity. Only β-hemolysis is considered harmful; as a result, two of the strains, namely BP20.9 and BP15.4, must not be considered potential probiotics. In this study, five strains (Py2.3, PY6.4, BP20.15, BP15.1, and BB10.1) showed γ-hemolytic activity, which indicates their safety for humans. Furthermore, none of the tested strains exhibited DNase activity.

An important advantage of probiotics is their potential to lower the cholesterol level in the plasma, which can reduce the risk of cardiovascular diseases. Manson et al. (1992) estimated that reducing cholesterol by 1% can decrease the risk of coronary artery disease by 2–3% [49]. All of the tested strains assimilated cholesterol from the broth during the 24 h of incubation, but only 5 strains showed the ability to absorb cholesterol at over 30% (PY2.3, PG10.5, BP20.15, BB10.1, and BB19.21). Thus, regular, long-term consumption of probiotics containing bee pollen and/or bee bread or supplementation of our diet with pure cultures of probiotic strains isolated from these products can positively affect the level of cholesterol in the plasma of consumers.

A significant amount of metabolized carbohydrates (mono-, di- and polysaccharides, glycosides, and triols) by tested *Bacillus* isolates prove that the investigated isolates can easily obtain carbon from various sources and can easily develop in the gut environment; moreover, the robust metabolism of carbohydrates can increase the viability of probiotics and can provide many different benefits to the host that include a positive effect on metabolic disease, improvement of the gut microbiome, and strengthening of the immune system [50, 51]. Taking into account the presence of probiotic bacteria in bee bread and bee pollen and the chemical composition of these products (high concentrations of saccharides), we suggest that both of them should be considered as symbiotics. According to the obtained results, three out of ten strains—PY2.3, BP20.15, and BB10.1—were considered possible probiotic strains. For these strains, whole-genome sequencing was performed. Two strains, BP20.15 and BB10.1, were classified as *B. subtilis* and PY2.3 as *B. velezensis*. Several studies showed that both species have great probiotic potential due to their survivability in the GIT, non-toxicity to the organism, and improvement of the health of the host [52–56]. All three isolates are potential producers of different antimicrobial compounds, including bacteriocins and secondary metabolites, which is an important benefit from the point of view of using these strains as probiotics.

## Conclusions

The preliminary outcomes of this study confirm the probiotic potential of some *Bacillus* spp. strains isolated from bee pollen or bee bread. On the other hand, we have found that some strains exhibit highly unfavorable properties, e.g., the ability of beta hemolysis or non-tolerance gut environment conditions, mostly low pH. Evidently, additional in vitro and in vivo tests are necessary to verify the possibility of using these isolates as probiotics (e.g., as ingredients in food products or dietary supplements) and to determine their positive impact on consumers’ health.

## Supplementary Information

Below is the link to the electronic supplementary material.

Supplementary file1 (DOCX 18 KB)

Supplementary file2 (DOCX 14 KB)

Supplementary file3 (PDF 767 KB)

## Data Availability

The data presented in this study are available on request from the corresponding author.
