# Supplementary material for: Probiotic potential of Bacillus Isolates from Polish Bee Pollen and Bee Bread
Source: Probiotics Antimicrob Proteins. 2023 Sep 19;17(1):364–77. doi: 10.1007/s12602-023-10157-4 (PMC11832673; doi:10.1007/s12602-023-10157-4)
Supplement: Supplementary file 1 — Supplementary file1 (DOCX 18 KB) [file 12602_2023_10157_MOESM1_ESM.docx]

**SUPPLEMENTARY MATERIALS**

Table 1. Carbohydrates metabolism of isolates derived from BP and BB. (+) represent particular carbohydrate hydrolysis after 24 and 48 hours of incubation.

|  | **PY2.3** | **PY5.3** | **PY6.4** | **PG10.5** | **BP20.9** | **BP20.15** | **BP15.4** | **BP15.1** | **BB10.1.2** | **BB19.21** |
| --- | --- | --- | --- | --- | --- | --- | --- | --- | --- | --- |
| **GLYCEROL** | **+** | **+** | **+** | **+** | **+** | **+** |  | **+** | **+** | **+** |
| **ERYTHRITOL** |  |  |  |  |  |  |  |  |  |  |
| **D-ARABINOSE** |  |  |  |  |  |  |  |  |  |  |
| **L-ARABINOSE** | **+** | **+** | **+** | **+** | **+** | **+** | **+** | **+** | **+** | **+** |
| **D-RIBOSE** |  | **+** | **+** | **+** | **+** | **+** | **+** | **+** | **+** | **+** |
| **D-XYLOSE** | **+** | **+** |  | **+** |  | **+** |  | **+** | **+** | **+** |
| **L-XYLOSE** |  |  |  |  |  |  |  |  |  |  |
| **D-ADONITOL** |  |  |  |  |  |  |  |  |  |  |
| **METHYL-β-D-XYLOPYRANOSIDE** |  |  |  |  |  |  |  |  |  |  |
| **D-GALACTOSE** |  |  |  | **+** | **+** |  |  | **+** |  | **+** |
| **D-GLUCOSE** | **+** | **+** | **+** | **+** | **+** | **+** | **+** | **+** | **+** | **+** |
| **D-FRUCTOSE** | **+** | **+** | **+** | **+** | **+** | **+** | **+** | **+** | **+** | **+** |
| **D-MANNOSE** | **+** | **+** | **+** | **+** | **+** | **+** | **+** | **+** | **+** | **+** |
| **L-SORBOSE** |  |  |  |  |  |  |  |  |  |  |
| **L-RHAMNOSE** |  |  |  |  |  |  |  |  |  |  |
| **DULCITOL** |  |  |  |  |  |  |  |  |  |  |
| **INOSITOL** | **+** |  |  |  |  | **+** |  | **+** | **+** |  |
| **D-MANNITOL** | **+** | **+** | **+** | **+** | **+** | **+** | **+** | **+** | **+** | **+** |
| **D-SORBITOL** | **+** | **+** | **+** |  |  | **+** |  | **+** | **+** |  |
| **METHYL-α-D-MANNOPYRANOSIDE** |  |  |  |  |  |  |  |  |  |  |
| **METHYL-α-D-GLUCOPYRANOSIDE** | **+** | **+** | **+** |  |  | **+** |  | **+** | **+** |  |
| **N-ACETYL-GLUCOSAMINE** |  |  | **+** |  |  |  | **+** | **+** |  |  |
| **AMYGDALIN** | **+** | **+** | **+** | **+** | **+** | **+** | **+** | **+** | **+** |  |
| **ARBUTIN** | **+** | **+** | **+** | **+** | **+** | **+** | **+** | **+** | **+** | **+** |
| **ESCULIN** | **+** | **+** | **+** | **+** | **+** | **+** | **+** | **+** | **+** | **+** |
| **SALICIN** | **+** | **+** | **+** | **+** | **+** | **+** | **+** | **+** | **+** | **+** |
| **D-CELLOBIOSE** | **+** | **+** | **+** | **+** | **+** | **+** | **+** | **+** | **+** | **+** |
| **D-MALTOSE** | **+** | **+** | **+** |  |  | **+** |  | **+** | **+** |  |
| **D-LACTOSE** | **+** | **+** | **+** |  |  |  |  |  | **+** |  |
| **D-MELIBIOSE** | **+** |  |  |  |  | **+** |  |  | **+** |  |
| **D-SACCHAROSE** | **+** | **+** | **+** | **+** | **+** | **+** | **+** | **+** | **+** | **+** |
| **D-TREHALOSE** | **+** | **+** | **+** | **+** | **+** | **+** | **+** | **+** | **+** | **+** |
| **INULIN** |  |  | **+** |  |  | **+** |  | **+** |  |  |
| **D-MELEZITOSE** |  |  |  |  |  |  |  |  |  |  |
| **D-RAFFINOSE** | **+** | **+** |  |  |  | **+** |  | **+** | **+** |  |
| **AMIDON** | **+** | **+** | **+** |  |  | **+** |  | **+** | **+** |  |
| **GLYCOGEN** | **+** | **+** | **+** |  |  | **+** |  | **+** | **+** |  |
| **XYLITOL** |  |  |  |  |  |  |  |  |  |  |
| **GENTIBIOSE** |  |  |  | **+** | **+** |  |  |  |  |  |
| **D-TURANOSE** |  |  | **+** |  |  | **+** |  | **+** |  |  |
| **D-LYXOSE** |  |  |  |  |  |  |  |  |  |  |
| **D-TAGATOSE** |  |  | **+** | **+** | **+** |  | **+** | **+** |  | **+** |
| **D-FUCOSE** |  |  |  |  |  |  |  |  |  |  |
| **L-FUCOSE** |  |  |  |  |  |  |  |  |  |  |
| **D-ARABITOL** |  |  |  |  |  |  |  |  |  |  |
| **L-ARABITOL** |  |  |  |  |  |  |  |  |  |  |
| **POTASSIUM GLUCONATE** |  |  |  |  |  |  |  |  |  |  |
| **POTASSIUM 2-KETOGLUCONATE** |  |  |  |  |  |  |  |  |  |  |
| **POTASSIUM 5-KETOGLUCONATE** |  |  |  |  |  |  |  |  |  |  |
