# Supplementary material for: Probiotic potential of Bacillus Isolates from Polish Bee Pollen and Bee Bread
Source: Probiotics Antimicrob Proteins. 2023 Sep 19;17(1):364–77. doi: 10.1007/s12602-023-10157-4 (PMC11832673; doi:10.1007/s12602-023-10157-4)
Supplement: Supplementary file 2 — Supplementary file2 (DOCX 14 KB) [file 12602_2023_10157_MOESM2_ESM.docx]

**Table S2. QUAST report summary**

| **BB10.1 Assembly** | |
| --- | --- |
| # contigs (>= 0 bp) | 45 |
| # contigs (>= 1000 bp) | 15 |
| # contigs (>= 5000 bp) | 13 |
| # contigs (>= 10000 bp) | 11 |
| # contigs (>= 25000 bp) | 9 |
| # contigs (>= 50000 bp) | 9 |
| Total length (>= 0 bp) | 4057020 |
| Total length (>= 1000 bp) | 4045914 |
| Total length (>= 5000 bp) | 4041831 |
| Total length (>= 10000 bp) | 4023237 |
| Total length (>= 25000 bp) | 3995073 |
| Total length (>= 50000 bp) | 3995073 |
| # contigs | 24 |
| Largest contig | 1057751 |
| Total length | 4051332 |
| GC (%) | 43.67 |
| N50 | 1041549 |
| N90 | 208390 |
| auN | 803853.6 |
| L50 | 2 |
| L90 | 6 |
| # N's per 100 kbp | 0 |
| **BP20.15 Assembly** | |
| # contigs (>= 0 bp) | 89 |
| # contigs (>= 1000 bp) | 48 |
| # contigs (>= 5000 bp) | 42 |
| # contigs (>= 10000 bp) | 39 |
| # contigs (>= 25000 bp) | 29 |
| # contigs (>= 50000 bp) | 22 |
| Total length (>= 0 bp) | 4030131 |
| Total length (>= 1000 bp) | 4018883 |
| Total length (>= 5000 bp) | 4004809 |
| Total length (>= 10000 bp) | 3977540 |
| Total length (>= 25000 bp) | 3826191 |
| Total length (>= 50000 bp) | 3588459 |
| # contigs | 53 |
| Largest contig | 503370 |
| Total length | 4022011 |
| GC (%) | 43.71 |
| N50 | 185768 |
| N90 | 42976 |
| auN | 210475.7 |
| L50 | 7 |
| L90 | 23 |
| # N's per 100 kbp | 0 |
| **PY2.3 Assembly** | |
| # contigs (>= 0 bp) | 42 |
| # contigs (>= 1000 bp) | 25 |
| Total length (>= 0 bp) | 3915986 |
| Total length (>= 1000 bp) | 3906247 |
| # contigs | 35 |
| Largest contig | 673476 |
| Total length | 3913508 |
| Reference length | 4065174 |
| GC (%) | 46.52 |
| Reference GC (%) | 46.35 |
| N50 | 353859 |
| NG50 | 353859 |
| N75 | 205761 |
| NG75 | 191619 |
| L50 | 4 |
| LG50 | 4 |
| L75 | 8 |
| LG75 | 9 |
| # misassemblies | 55 |
| # misassembled contigs | 11 |
| Misassembled contigs length | 3390995 |
| # local misassemblies | 31 |
| # scaffold gap ext. mis. | 0 |
| # scaffold gap loc. mis. | 0 |
| # unaligned mis. contigs | 0 |
| # unaligned contigs | 4 + 11 part |
| Unaligned length | 222589 |
| Genome fraction (%) | 91.819 |
| Duplication ratio | 1.001 |
| # N's per 100 kbp | 0 |
| # mismatches per 100 kbp | 1218 |
| # indels per 100 kbp | 34.16 |
| Largest alignment | 261666 |
| Total aligned length | 3690168 |
| NA50 | 94176 |
| NGA50 | 89223 |
| NA75 | 44598 |
| NGA75 | 39451 |
| LA50 | 12 |
| LGA50 | 13 |
| LA75 | 28 |
| LGA75 | 31 |
