# Supplementary material for: Probiotic potential of Bacillus Isolates from Polish Bee Pollen and Bee Bread
Source: Probiotics Antimicrob Proteins. 2023 Sep 19;17(1):364–77. doi: 10.1007/s12602-023-10157-4 (PMC11832673; doi:10.1007/s12602-023-10157-4)
Supplement: Supplementary file 3 — Supplementary file3 (PDF 767 KB) [file 12602_2023_10157_MOESM3_ESM.pdf]

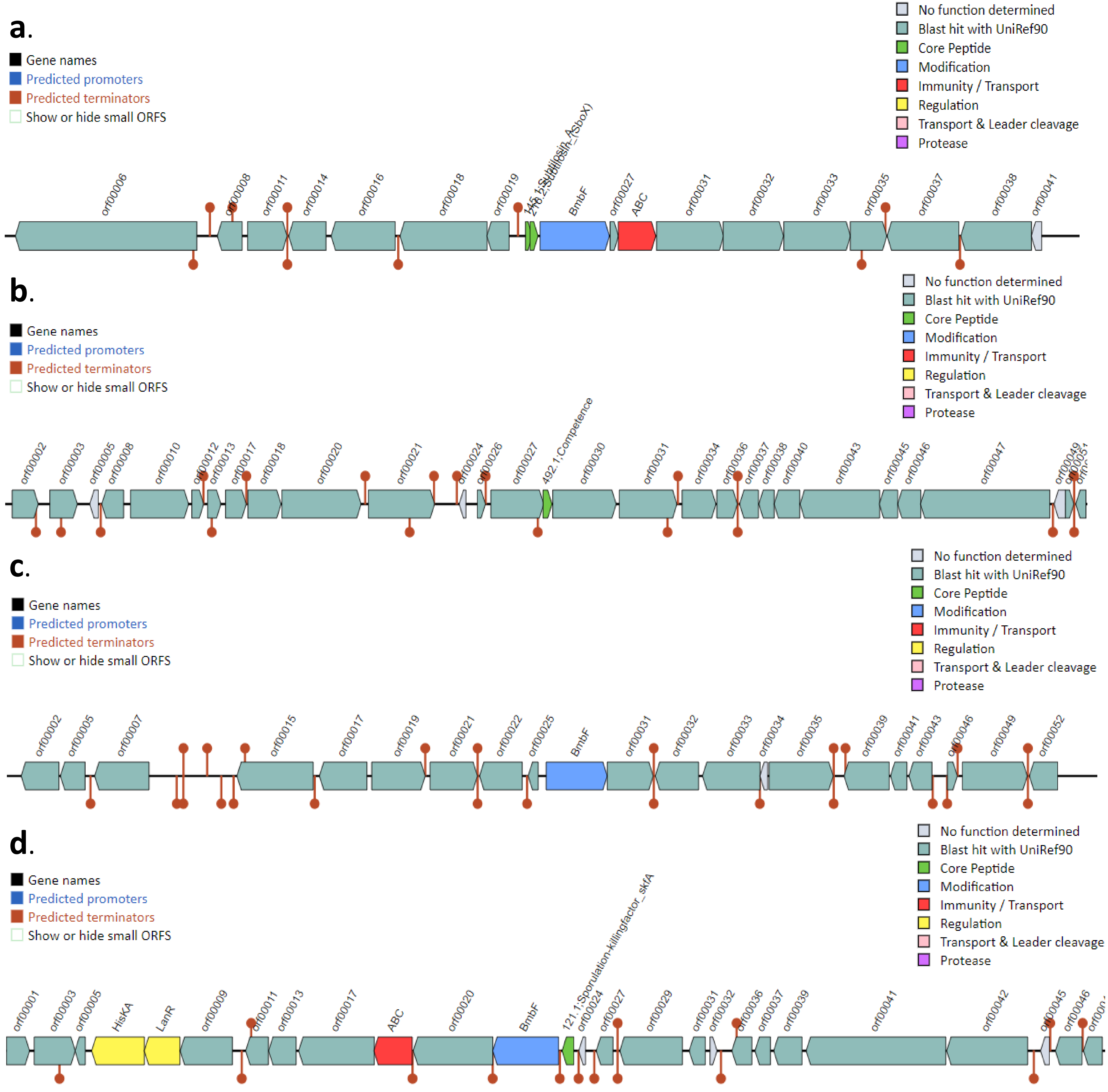

Figure S1. The organization of bacteriocin gene clusters in the isolate *B. subtilis* 20.15 genome predicted through the BAGEL4 webserver. The area of interest represents (a) Subtilosin (b) competence (c) sactipeptides classes (d) sporulation killing factor. The color schemes represent the specific gene clusters identified in BP 20.15 genome.

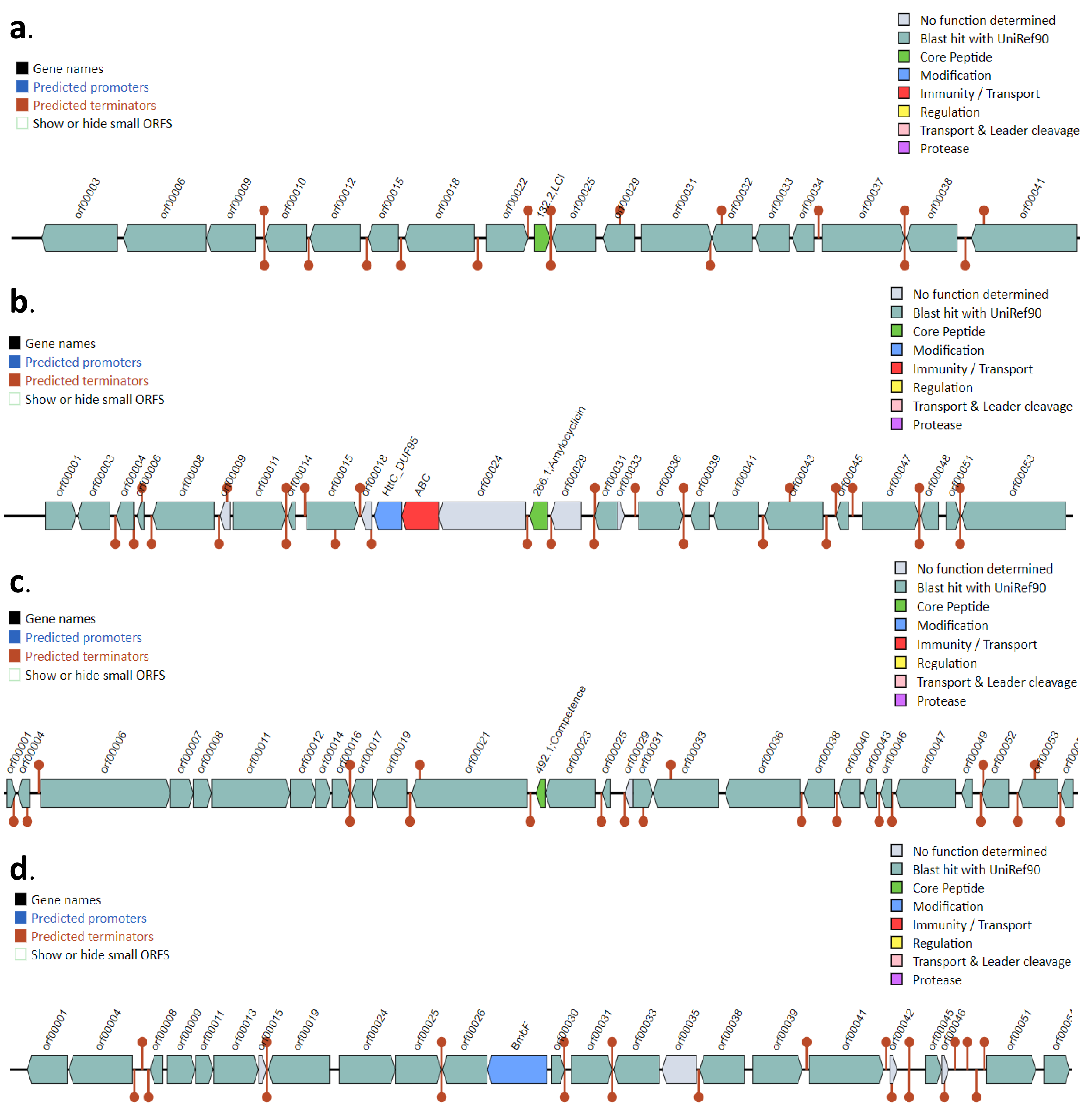

Figure S2. The organization of bacteriocin gene clusters in the isolate *B. velezensis* PY2.3 genome predicted through the BAGEL4 webserver. The area of interest represents (a) AMP LCI (b) Amylocyclicin (c) competence (d) sactipeptide classes. The color schemes represent the specific gene clusters identified in PY2.3 genome.
